# Integrated transcriptomic and single-cell analyses define neuronal heterogeneity and PDIA4-associated malignancy in glioblastoma

**DOI:** 10.3389/fonc.2026.1971414

**Published:** 2026-10-13

**Authors:** Zhixin Liu, Xiaojuan Luo, Xuetao Deng, Yihao Liu, Wenrui Wu

**Affiliations:** 1 Nanchang County People’s Hospital, Nanchang, Jiangxi, China; 2 Department of Neurosurgery, Xiangyang Central Hospital, Affiliated Hospital of Hubei University of Arts and Science, Xiangyang, China; 3 Department of Neurosurgery, The First Affiliated Hospital, Fujian Medical University, Fuzhou, Fujian, China; 4 Department of Geriatrics, Xiangyang Central Hospital, Affiliated Hospital of Hubei University of Arts and Science, Xiangyang, China; 5 Department of Neurosurgery, The First Affiliated Hospital, Jiangxi Medical College, Nanchang University, Nanchang, Jiangxi, China

**Keywords:** glioblastoma, microenvironment, neuronal subpopulation, PDIA4, prognostic model, single-cell RNA

## Abstract

**Background:**

Glioblastoma (GBM) is characterized by extensive cellular heterogeneity and complex interactions between malignant cells and the surrounding microenvironment. The contribution of neuronal populations to GBM progression and prognosis, however, remains incompletely understood.

**Methods:**

Bulk transcriptomic data from TCGA-GBM and GTEx were integrated with two single-cell RNA-seq datasets. Differential expression analysis, WGCNA, machine-learning-based survival modeling, CIBERSORT, GSEA, CopyKAT, MiloR, pseudotime analysis, GSVA, CellChat, and pySCENIC were used to characterize prognostic genes and their cellular context. PDIA4 was further examined in U251 and U87 cells using siRNA-mediated knockdown followed by Western blotting, qPCR, Transwell invasion, wound-healing, colony-formation, and CCK-8 assays.

**Results:**

Nine prognostic genes—PDIA4, CAMK2N1, RPH3A, RAB11FIP4, DMTN, EPHB6, SLC47A2, ESM1, and EMILIN2—were retained in the final prognostic model. Single-cell analysis showed distinct cellular distributions of these genes and identified a tumor-associated neuronal population enriched in GBM. Pseudotime analysis revealed transcriptional differences between normal- and tumor-associated neuronal states, while CellChat predicted altered NRXN–NLGN communication involving neuronal populations and GBM cells. Immune deconvolution showed associations between several prognostic genes and immune-cell composition, including an inverse correlation between PDIA4 and activated NK cells. Experimentally, PDIA4 was expressed at higher levels in U251 and U87 cells than in SVG p12 cells. PDIA4 knockdown reduced clonogenic growth, invasion, wound closure, and CCK-8 metabolic activity readouts in both GBM cell lines.

**Conclusions:**

Integrative bulk and single-cell analyses linked GBM prognosis to distinct neuronal and immune features of the tumor microenvironment. Together, these analyses place the nine-gene prognostic signature within the cellular architecture of GBM and highlight tumor-associated neuronal states as a potential component of the prognostic tumor ecosystem. PDIA4 knockdown provided complementary functional support for one component of the signature, while the predicted NRXN–NLGN network nominates a candidate route of neuron–GBM communication for future investigation.

## 1 Introduction

Glioblastoma (GBM) is an aggressive primary brain tumor characterized by extensive cellular heterogeneity, diffuse infiltration, and resistance to conventional therapy. Increasing evidence indicates that GBM progression is shaped not only by malignant-cell-intrinsic programs but also by interactions with immune, vascular, glial, and neuronal components of the tumor microenvironment (1–3). Single-cell studies have further shown that both malignant and non-malignant cell states undergo substantial remodeling during tumor progression and treatment (1, 2). These findings highlight the importance of interpreting prognostic molecular alterations within their cellular context.

The contribution of neuronal components to GBM biology has attracted particular attention. Glioma cells can exploit neuronal programs involved in migration and network formation and can receive functional synaptic input from surrounding neurons (4). Experimental and clinical studies have shown that neuronal activity promotes glioma growth and invasion, while stronger tumor–brain functional connectivity is associated with poorer patient survival (4, 5). GBM cells can also form electrically active tumor networks that support tumor expansion (6). More recently, neuronal circuit remodeling has been linked to regional immunosuppression within GBM, suggesting that neural and immune features of the microenvironment may be functionally interconnected (7). These observations provide a rationale for examining neuronal heterogeneity and neuron-associated signaling in GBM at single-cell resolution.

Integration of bulk and single-cell transcriptomic data offers an opportunity to connect prognostic signals with their cellular origins. Bulk RNA sequencing provides sufficient sample size for differential expression and survival modeling, whereas single-cell analysis can identify the specific cell populations contributing to these signals. Recent studies have demonstrated that GBM ecosystems contain reproducible combinations of malignant states and microenvironmental populations and that these states can change substantially during disease evolution (1, 2). Combining these complementary approaches may therefore help distinguish broadly expressed prognostic markers from genes associated with specific cellular states or microenvironmental interactions.

PDIA4, an endoplasmic-reticulum-associated protein involved in protein folding and stress responses, has been implicated in GBM progression. Previous studies showed that PDIA4 is upregulated in GBM and promotes tumor-cell proliferation through PI3K/AKT/mTOR signaling (8). PDIA4 has also been linked to endoplasmic-reticulum stress, VEGFA production, angiogenesis, and resistance to anti-angiogenic therapy (9). However, its relationship with the cellular architecture of the GBM microenvironment remains incompletely understood.

In this study, we integrated bulk RNA-seq, weighted gene co-expression network analysis, machine-learning-based prognostic modeling, immune deconvolution, and single-cell transcriptomics to characterize prognostic and microenvironmental features of GBM. Prognostic genes were mapped to individual cell populations, followed by analyses of neuronal differential abundance, pseudotime, pathway activity, cell–cell communication, and transcription-factor regulation. PDIA4 was further selected for *in vitro* functional validation. This strategy aimed to link prognostic molecular signatures with their cellular context and to characterize neuronal and immune features associated with GBM progression.

## 2 Materials and methods

### 2.1 Data acquisition and differential expression analysis

Single-cell RNA-seq datasets GSE237673 and GSE273274 were obtained from the Gene Expression Omnibus. After quality control, 93,206 cells were retained. Bulk RNA-seq data included 311 TCGA-GBM samples and 173 non-cancer brain samples from GTEx donors from GTEx. The combined TCGA/GTEx dataset was used for differential expression analysis, whereas the TCGA cohort was randomly divided into training and validation sets at a 7:3 ratio for prognostic modeling. Differentially expressed genes were identified using DESeq2 (1.48.2) with |log2FC| > 3 and *P* < 0.05.

### 2.2 WGCNA

Batch effects were adjusted using the ComBat function in the `sva` package. The 5,000 genes with the highest median absolute deviation were included in WGCNA. The soft-thresholding power was set to 3 based on the scale-free topology criterion (R² > 0.85). An unsigned network was constructed with a minimum module size of 30. Module eigengenes were correlated with tumor status using Pearson correlation. Genes from GBM-associated modules were intersected with the differentially expressed genes, and 306 overlapping genes from the turquoise module were retained for further analysis.

### 2.3 Prognostic model construction

Candidate genes were initially screened by univariable Cox regression. Prognostic models were generated using the Mime1 framework, integrating LASSO, Ridge, Elastic Net, Random Survival Forest, CoxBoost, Stepwise Cox, plsRcox, SuperPC, and combined algorithms. Five-fold cross-validation and 100 bootstrap resamplings were used to evaluate model performance. The StepCox[forward] + RSF model was selected according to its overall C-index and survival-prediction performance. Nine genes—PDIA4, CAMK2N1, RPH3A, RAB11FIP4, DMTN, EPHB6, SLC47A2, ESM1, and EMILIN2—were retained for construction of the final prognostic model and nomogram.

### 2.4 Functional enrichment and immune infiltration analyses

Single-gene enrichment analysis was performed for the nine prognostic genes using GO and KEGG gene sets obtained from `msigdbr`, with gene-set processing performed using `GSEABase`. Immune-cell fractions were estimated using CIBERSORT with the LM22 signature matrix. Each bulk RNA-seq sample was treated as an independent biological observation, and differences in estimated immune-cell fractions between tumor and normal samples were evaluated using the Wilcoxon rank-sum test. Individual inferred immune-cell fractions were not treated as independent replicates. Associations between prognostic-gene expression and immune-cell abundance were assessed using Spearman correlation, with |r| > 0.30 and P < 0.01 considered significant.

### 2.5 Single-cell processing and annotation

Single-cell data were processed in Seurat (5.3.0). Cells with 200–7,000 detected genes and mitochondrial RNA content <15% were retained. Principal component analysis was performed, and the first 30 components were used for downstream analysis. Batch effects were corrected using Harmony (1.2.3). A K-nearest-neighbor graph was constructed with k = 40, followed by Louvain clustering at a resolution of 0.1. Cluster markers were identified using FindAllMarkers with log2FC > 0.25, min.pct > 0.25, and FDR < 0.05. Cell types were assigned according to canonical markers. The integrated single-cell dataset comprised 93,206 cells from 17 biological samples from GSE237673 and GSE273274. Cell-type proportions were calculated separately for each sample to examine inter-sample variation. Cells from the same sample were not considered independent biological replicates in abundance comparisons.

### 2.6 Copy-number and neuronal differential-abundance analyses

Copy-number alterations were inferred from scRNA-seq expression profiles using CopyKAT (v1.1.0) and therefore did not constitute an independent genomic data layer. Aneuploid cells were classified as malignant GBM cells, whereas diploid cells were considered non-malignant. Neuronal cells were subsequently extracted for differential-abundance analysis using miloR (v1.0.0). A K-nearest-neighbor graph was constructed with k = 15, and neighborhoods were defined using makeNhoods with d = 30. Neighborhood cell counts were summarized according to biological sample, and differential abundance was tested using the tumor/normal group assignment (design = ~Group) rather than treating individual cells as independent replicates. Differentially abundant neighborhoods were identified using testNhoods, with FDR < 0.1 considered significant. Based on the direction and distribution of differential abundance, neuronal populations were subsequently categorized as Normal_neurons, Tumour_neurons, Special_neurons, or Unrelated_neurons for downstream analyses.

### 2.7 Pseudotime and GSVA analyses

Transcriptional relationships among neuronal cells were explored using Monocle2 (v2.24.1) with the DDRTree dimensionality-reduction algorithm. Cells were ordered along the inferred pseudotime manifold using orderCells. BEAM analysis was performed at the selected bifurcation point (branch point 2) to identify genes showing differential expression across the inferred branches. The pseudotime analysis was used to characterize relationships among transcriptional states and was not interpreted as evidence of lineage conversion. Pathway activity among cell populations was assessed by GSVA using the ssGSEA method. GO biological-process and KEGG gene sets were obtained from msigdbr (v7.5.1), and pathway differences were evaluated using limma with |log2FC| > 0.5 and FDR < 0.05.

### 2.8 Cell–cell communication and transcription-factor analyses

Cell–cell communication was analyzed using CellChat (v1.1.3). Separate CellChat objects were constructed for tumor and normal samples using the human ligand–receptor interaction database. Communication probabilities and pathway-level information flow were compared between groups, with particular attention to NRXN signaling. Transcription-factor regulatory networks were inferred using pySCENIC. Gene regulatory relationships were generated with grnboost2, followed by motif enrichment using ctx. Regulon activity was quantified using AUCell, and selected transcription factors and their predicted downstream targets were visualized in R. Because CellChat infers ligand-receptor communication from transcriptomic data, the resulting interactions were interpreted as candidate communication events and not as evidence of protein expression, receptor activation, synaptic localization, or functional signaling.

### 2.9 Cell culture and reagents

U251 and U87 human glioma cells and SVG p12 cells were obtained from Servicebio (Wuhan, China) and cultured under the recommended conditions. Cells were maintained at 37 °C in a humidified atmosphere containing 5% CO_2_ and were used at low passage (approximately passages 5–20 after recovery). Cultures were routinely monitored for mycoplasma contamination and were negative during the experimental period. Independent STR re-authentication was not performed in the present study. DMEM, FBS, 4% paraformaldehyde, and crystal violet were obtained from Solarbio (Beijing, China). PDIA4 siRNA and qPCR primers were synthesized by Tsingke Biotechnology (Beijing, China). Transwell inserts and Matrigel were purchased from Corning (New York, NY, USA). qPCR reagents were obtained from Yeasen Biotechnology (Shanghai, China), and the CCK-8 kit was purchased from Shanghai Sheng’er Biotechnology Co., Ltd. (Shanghai, China). Antibodies against PDIA4 and GAPDH were obtained from Wuhan Sanying Biotechnology (Wuhan, China).

### 2.10 siRNA transfection

For siRNA-mediated knockdown, U251 and U87 cells were seeded at approximately 50-60% confluence and transfected with PDIA4-targeting siRNA or a non-targeting negative-control siRNA at a final concentration of 50 nM using Lipofectamine 3000 according to the manufacturer’s instructions. Cells were collected 48 h after transfection for qRT-PCR and Western blot analysis or used in the indicated functional assays.

The PDIA4 siRNA sequences were:

sense: 5′-GAGUCUUGGUCCUAAAUGA(dT)(dT)-3′;

antisense: 5′-UCAUUUAGGACCAAGACUC(dT)(dT)-3′.

### 2.11 Quantitative real-time PCR

Total RNA was extracted using TRIzol reagent and reverse-transcribed into cDNA. qPCR was performed using SYBR Green reagents according to the manufacturer’s protocol. GAPDH served as the internal reference, and relative PDIA4 expression was calculated using the 2^−ΔΔCt method.

The PDIA4 primers were:

forward: 5′-CCGCTAACAACCTGAGAGAAGAT-3′;

reverse: 5′-GGGCTCATACTTGGACTGGAATT-3′.

### 2.12 Western blotting

Total protein was extracted using RIPA lysis buffer supplemented with protease inhibitors, and protein concentrations were determined using a BCA assay. Equal amounts of protein 20−30μgperlane were separated by SDS-PAGE and transferred to PVDF membranes. Membranes were blocked with 5% non-fat milk for 1 h at room temperature and incubated overnight at 4 °C with primary antibodies against PDIA4 (1:1,000) and GAPDH (1:5,000), followed by the appropriate HRP-conjugated secondary antibodies (1:5,000). Signals were detected using enhanced chemiluminescence. Band intensity was quantified by ImageJ, and PDIA4 expression was normalized to GAPDH. Quantitative data were obtained from three independent biological experiments.

### 2.13 Transwell invasion, wound-healing, and colony-formation assays

For invasion assays, Matrigel-coated Transwell inserts with 8-μm pores were used. At 48 h after transfection, 5 × 10^4^ cells suspended in serum-free medium were seeded into the upper chamber, while medium containing 10% FBS was added to the lower chamber as a chemoattractant. After 24 h, cells remaining on the upper surface were removed, whereas cells that had traversed the membrane were fixed with 4% paraformaldehyde, stained with crystal violet, and counted microscopically. Three independent biological experiments were performed. For wound-healing assays, confluent monolayers were scratched with a sterile 200-μL pipette tip and maintained in serum-free medium to reduce, but not completely eliminate, the contribution of cell proliferation. Images were acquired at 0 and 48 h and wound closure was quantified using ImageJ. Because differences in cell growth may still influence wound closure, this assay was interpreted as a composite growth/motility phenotype rather than a migration-specific measurement.

### 2.14 CCK-8 assay

Transfected U251 and U87 cells were seeded in 96-well plates at approximately 2 × 10³ cells per well. CCK-8 reagent was added according to the manufacturer’s instructions, and absorbance at 450 nm was recorded at 0, 2, and 4 h after reagent addition. These time points represent the duration of color development rather than cell-culture time, and the assay was therefore interpreted as a measure of viable-cell metabolic activity.

### 2.15 Statistical analysis

Statistical analyses were performed using R and GraphPad Prism. Two-group comparisons were conducted using Student’s *t*-test or the Wilcoxon rank-sum test, as appropriate. Multiple-group comparisons were performed using one-way ANOVA or the Kruskal–Wallis test. Correlations were assessed by Spearman analysis, and the Benjamini–Hochberg method was applied for multiple-testing correction where required. *In vitro* experiments were performed independently at least three times unless otherwise stated. Data are presented as mean ± SD. Comparisons between two groups were performed using two-tailed unpaired Student’s t-test, whereas comparisons involving more than two groups were evaluated by one-way ANOVA followed by Tukey’s multiple-comparison test. P < 0.05 was considered statistically significant.

## 3 Results

### 3.1 Identification of GBM-associated genes

Differential expression analysis between TCGA-GBM and GTEx non-cancer donors brain samples identified 4,524 DEGs, including 2,999 upregulated and 1,525 downregulated genes (|log2FC| > 3, *P* < 0.05; **Figure 1A**). GO and KEGG analyses showed enrichment in pathways related to nervous system development, synaptic functions, small GTPase-mediated signaling, and metabolism (**Figure 1B**). WGCNA identified several co-expression modules associated with tumor status (**Figures 1C–E**). The turquoise and black modules showed significant associations with GBM and contained 2,549 and 338 genes, respectively. Functional enrichment indicated that the turquoise module was mainly related to neuronal and synaptic processes, whereas the black module was associated with DNA replication and chromosome organization (**Figure 1F**). Intersection with the DEGs yielded 306 genes from the turquoise module and 60 genes from the black module (**Figure 1G**). The 306 overlapping genes from the turquoise module were used for subsequent prognostic analysis.

**Figure 1:**
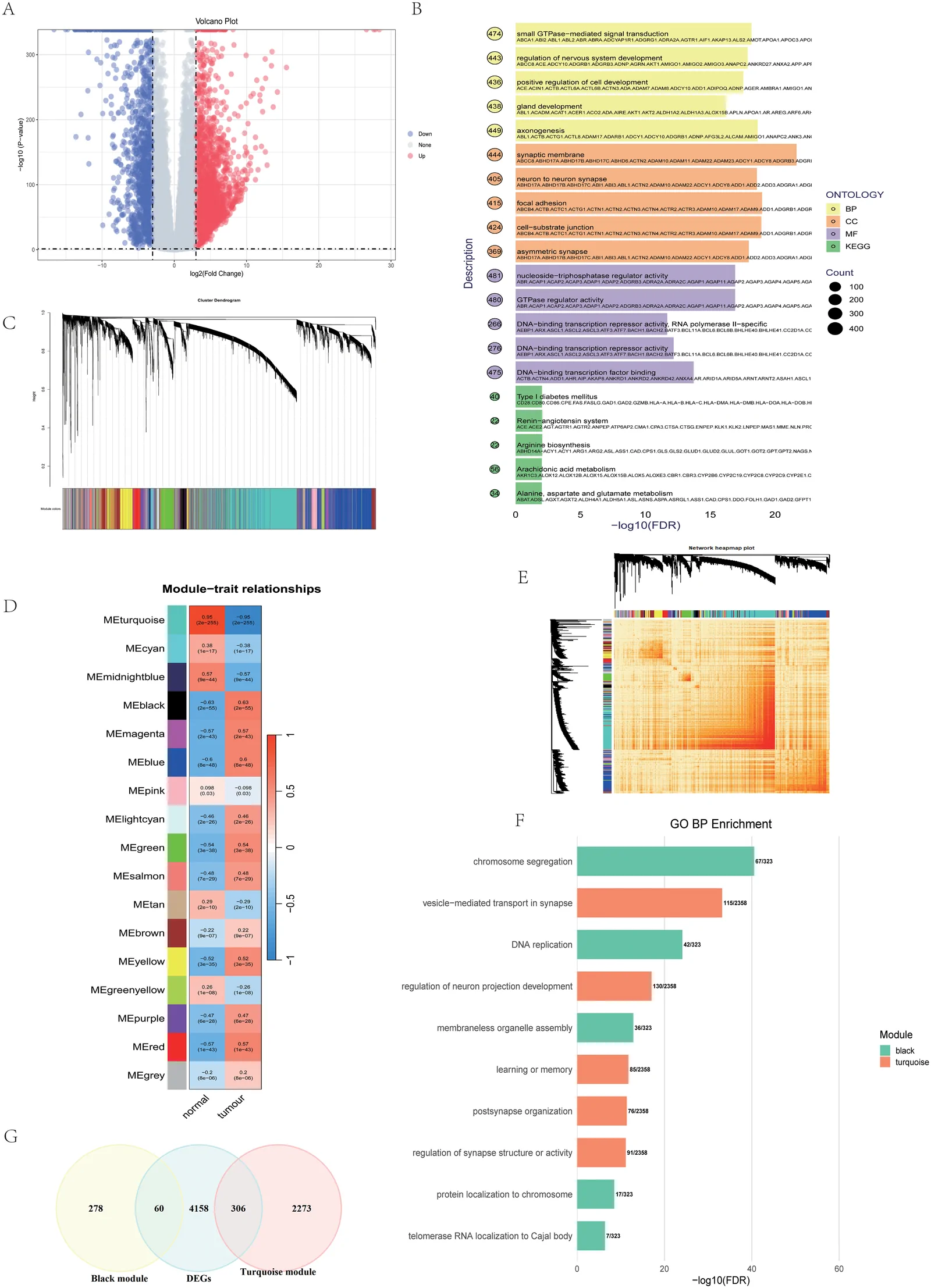
Differential expression analysis and identification of GBM-associated gene modules by WGCNA. **(A)** Volcano plot showing differentially expressed genes between GBM and normal brain tissues using |log2FC| > 3 and P < 0.05 as cutoffs. **(B)** GO and KEGG enrichment analyses of the differentially expressed genes. **(C)** Hierarchical clustering dendrogram of genes assigned to WGCNA modules; genes not assigned to a specific module are shown in gray. **(D)** Heatmap showing correlations between module eigengenes and clinical traits. **(E)** Eigengene adjacency heatmap showing relationships among the identified modules. **(F)** GO enrichment analysis of genes in the black and turquoise modules. **(G)** Overlap between differentially expressed genes and genes from the black and turquoise modules.

### 3.2 Construction of the prognostic model

Univariate Cox analysis and machine-learning modeling were performed using the candidate genes. Among 101 model combinations, StepCox[forward] + RSF showed the best overall performance in the training and validation cohorts (**Figure 2A**). The 1-year survival AUC was 0.938 in the training cohort and 0.678 in the validation cohort (**Figure 2B**). Nine genes—PDIA4, CAMK2N1, RPH3A, RAB11FIP4, DMTN, EPHB6, SLC47A2, ESM1, and EMILIN2—were retained in the final model and incorporated into the nomogram (**Figure 2C**). Calibration analysis showed agreement between predicted and observed survival probabilities (**Figure 2D**), and the model showed comparable predictive performance with previously established GBM models (**Figure 2E**). All nine genes were differentially expressed between tumor and normal samples. PDIA4, SLC47A2, ESM1, and EMILIN2 were upregulated in GBM, whereas the remaining genes were downregulated (**Figure 2F**).

**Figure 2:**
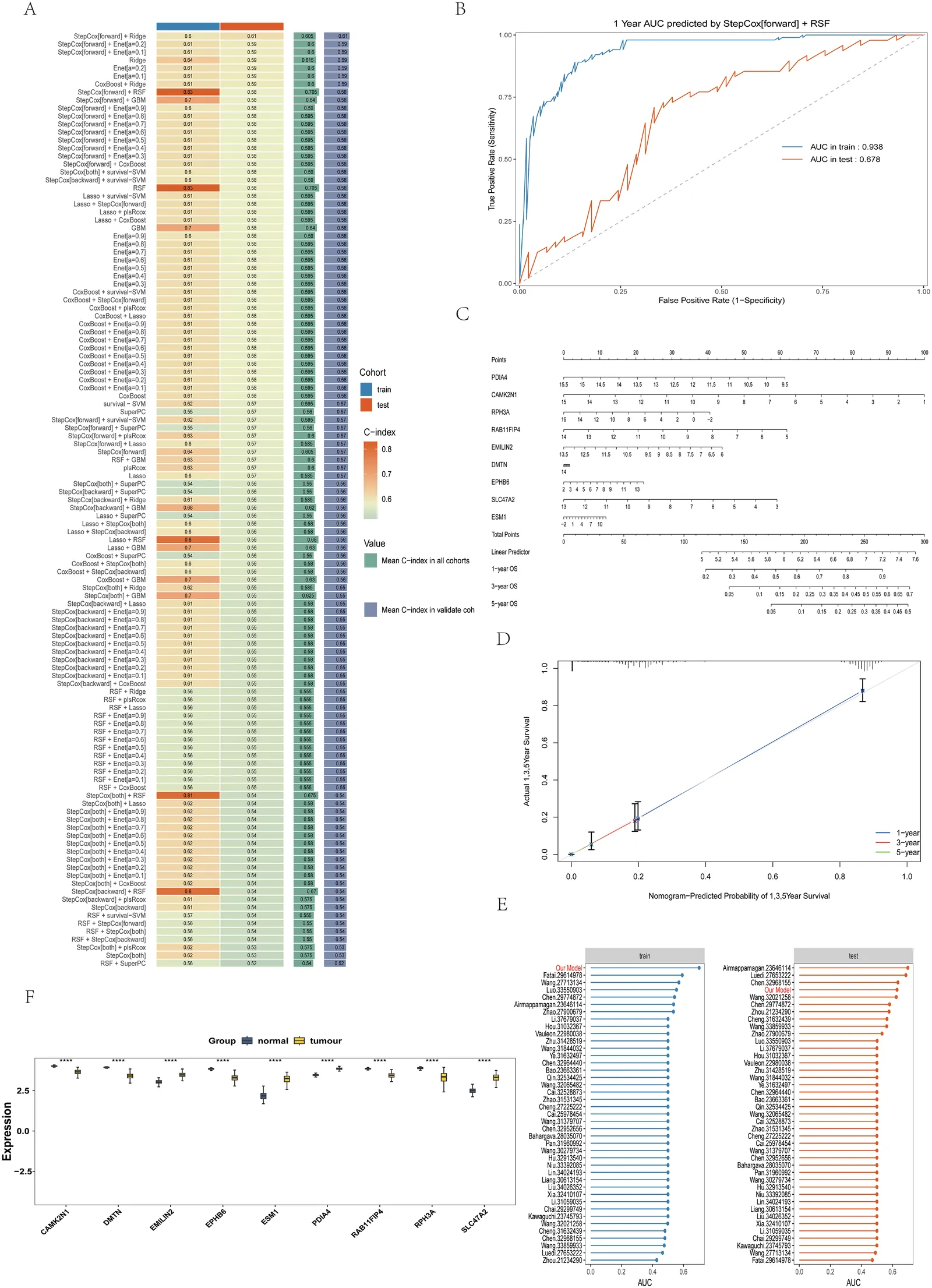
Construction and evaluation of the GBM prognostic model. **(A)** Heatmap of C-index values for 101 machine-learning model combinations in the training and validation cohorts. **(B)** Receiver operating characteristic curves for 1-year survival generated using the StepCox[forward] + RSF model in the training and validation cohorts. **(C)** Nomogram based on the nine genes retained in the final prognostic model. **(D)** Calibration curves comparing predicted and observed survival probabilities. **(E)** Comparison of the predictive performance of the present model with previously established GBM models included in the Mime1 package. **(F)** Expression of the nine prognostic genes—PDIA4, CAMK2N1, RPH3A, RAB11FIP4, DMTN, EPHB6, SLC47A2, ESM1, and EMILIN2—in tumor and normal samples.

### 3.3 Functional characteristics of the prognostic genes

Single-gene GSEA revealed distinct functional associations among the nine prognostic genes (**Figures 3A–I**). PDIA4, ESM1, SLC47A2, and EMILIN2 were mainly associated with immune-related processes, including antigen processing and presentation. RPH3A, RAB11FIP4, DMTN, and SLC47A2 were linked to neuronal and synaptic functions, whereas CAMK2N1 and EPHB6 showed enrichment in pathways related to DNA replication and chromosome organization. Overall, the signature genes were associated with immune regulation, cell proliferation, and neuronal signaling.

**Figure 3:**
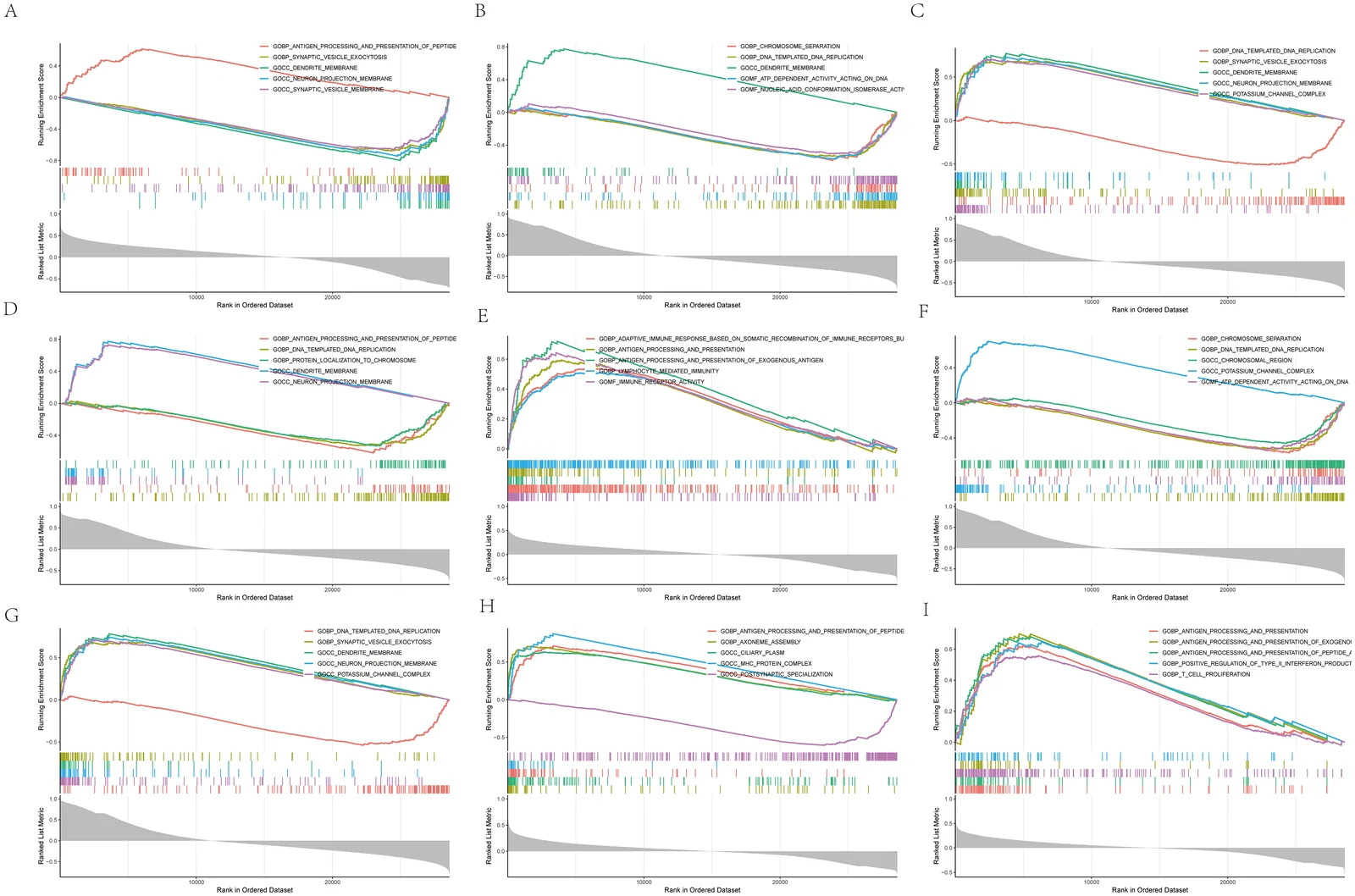
Gene set enrichment analysis of the nine prognostic genes. Single-gene GSEA showing the biological processes and pathways associated with **(A)** PDIA4, **(B)** CAMK2N1, **(C)** RPH3A, **(D)** RAB11FIP4, **(E)** DMTN, **(F)** EPHB6, **(G)** SLC47A2, **(H)** ESM1, and **(I)** EMILIN2. Enriched gene sets include pathways related to immune regulation, DNA replication and chromosome organization, and neuronal or synaptic functions.

### 3.4 Immune infiltration associated with prognostic genes

CIBERSORT analysis showed marked differences in immune-cell composition between tumor and normal tissues (**Figures 4A–D**). Significant changes were observed in M1 and M2 macrophages, resting and activated NK cells, mast cells, neutrophils, follicular helper T cells, and CD8 T cells. Among the tumor-upregulated prognostic genes, PDIA4 expression showed a negative association with the estimated fraction of activated NK cells and positively correlated with resting NK cells (**Figure 4E**). SLC47A2, EMILIN2, and ESM1 were also associated with several immune-cell populations, including macrophages, NK cells, monocytes, neutrophils, and mast cells (**Figures 4F–H**).

**Figure 4:**
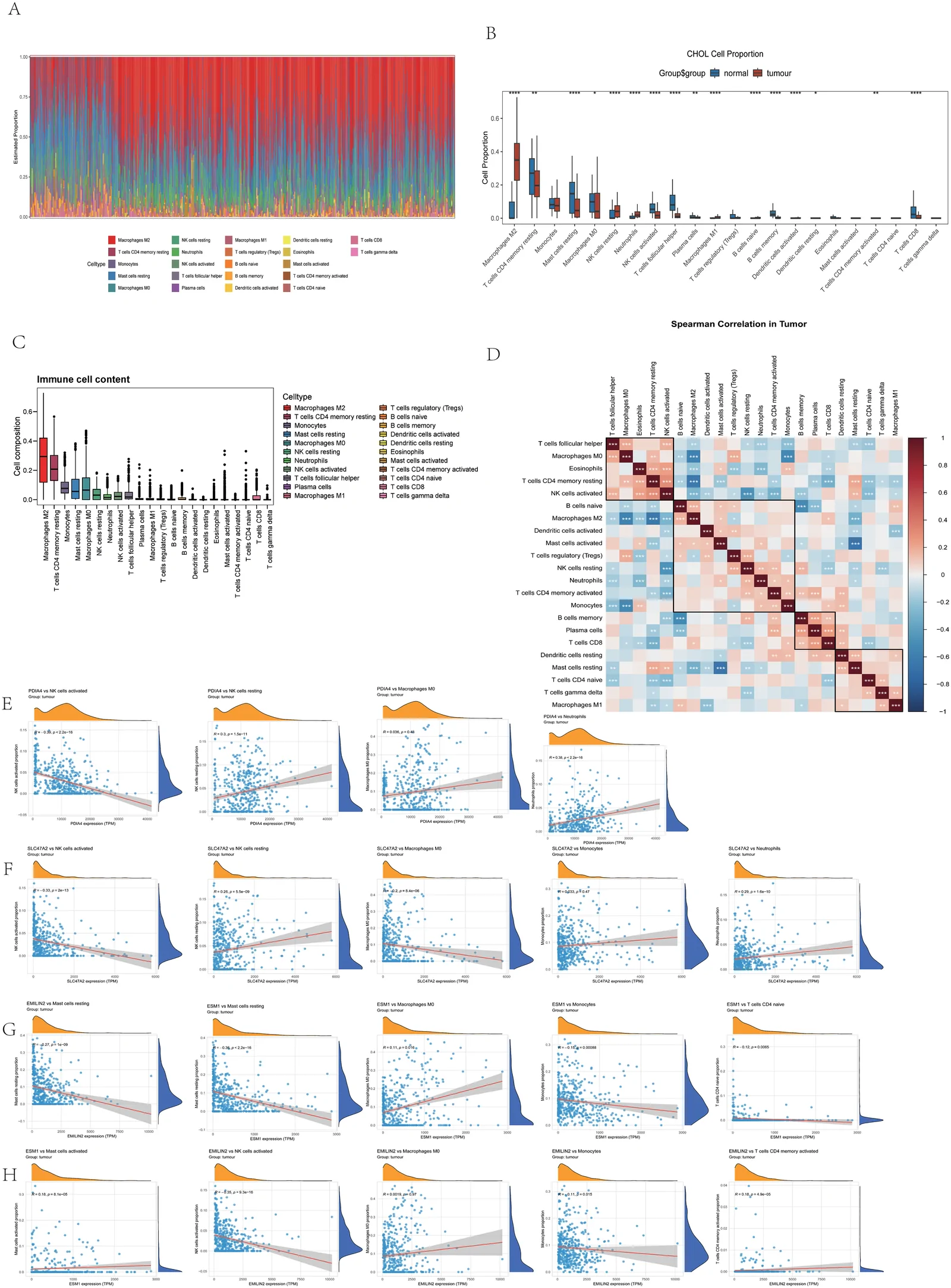
Immune-cell infiltration and its association with prognostic gene expression in GBM. **(A)** Relative proportions of 22 immune-cell populations estimated by CIBERSORT across individual samples. **(B)** Comparison of immune-cell abundance between tumor and normal groups. **(C)** Distribution of immune-cell fractions showing cell populations with significant differences between the two groups. **(D)** Correlation matrix of the estimated immune-cell populations. **(E–H)** Correlations between immune-cell abundance and the expression of PDIA4 **(E)**, SLC47A2 **(F)**, EMILIN2 **(G)**, and ESM1 **(H)**.

### 3.5 Single-cell characterization of the GBM microenvironment

CopyKAT identified 53,836 aneuploid cells, which were classified as GBM cells (**Figures 5A, E**). Following integration, dimensionality reduction, and annotation, the major cell populations included GBM cells, astrocytes, endothelial cells, lymphocytes, macrophages, microglia, mural cells, neurons, oligodendrocytes, and oligodendrocyte precursor-like cells (**Figures 5B, C, F, H**). The prognostic genes showed distinct cellular distributions (**Figure 5D**). ESM1 was mainly expressed in endothelial cells, EPHB6, DMTN, RAB11FIP4, and RPH3A were enriched in neuronal populations, and CAMK2N1 was predominantly expressed in microglia. GSVA further revealed cell-type-specific differences in neuronal, metabolic, and developmental pathways (**Figure 5G**). Although the overall composition differed between the tumor and normal groups, appreciable inter-sample heterogeneity was evident when cell-type proportions were examined separately for the 17 biological samples. We therefore used the sample identity, rather than individual cells, as the biological replication level for subsequent abundance analysis. (**Figures 5I, J**).

**Figure 5:**
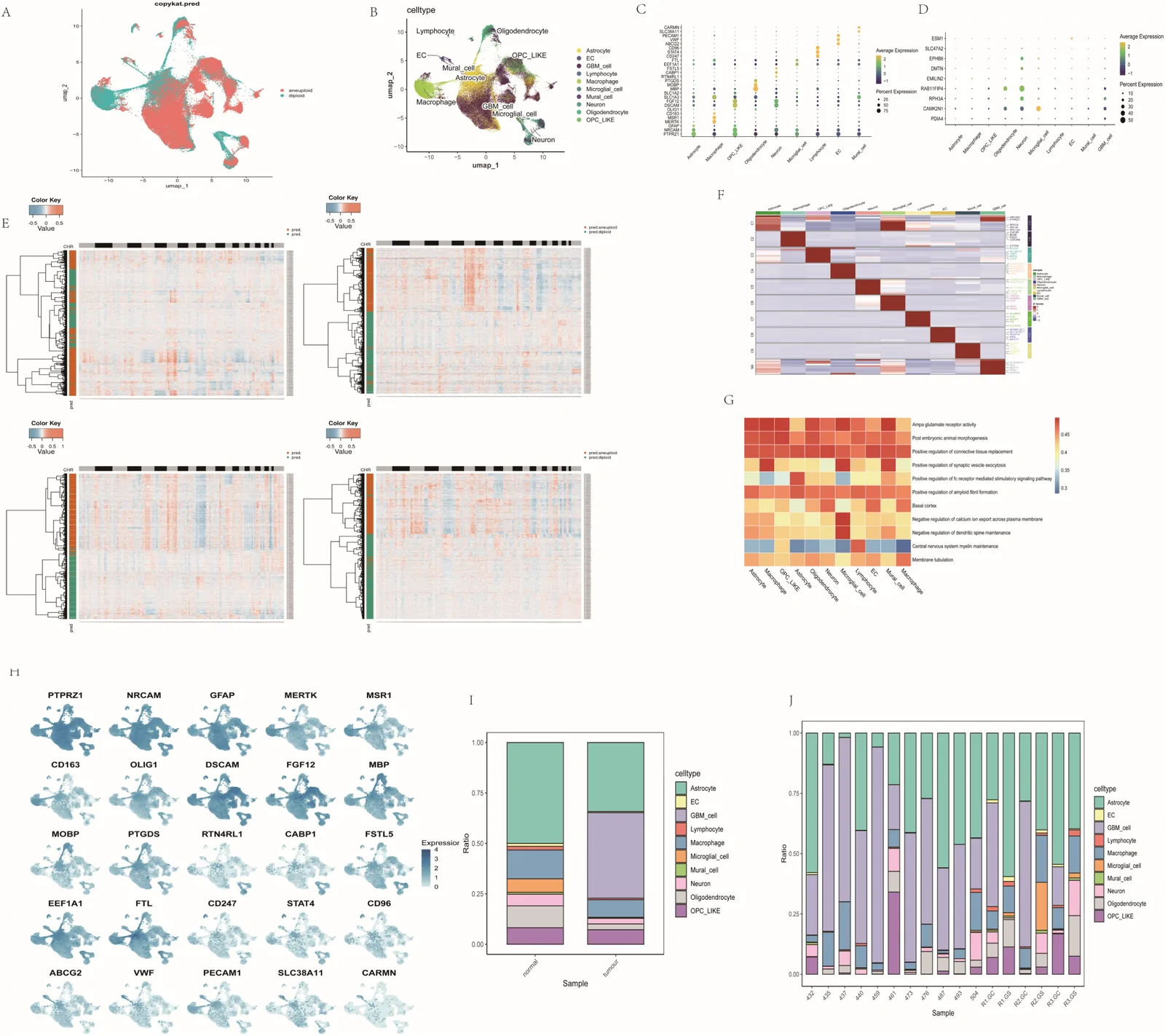
Single-cell identification and characterization of cell populations in the GBM microenvironment. **(A)** CopyKAT-based classification of aneuploid and diploid cells. **(B)** UMAP representation of the annotated cell populations in the integrated single-cell dataset. **(C)** Expression of canonical marker genes used for cell-type annotation. **(D)** Distribution of the nine prognostic genes across the identified cell populations. **(E)** CopyKAT heatmaps showing inferred copy-number profiles; the single-cell matrix was divided into four subsets for computational processing. **(F)** Heatmap of the top cell-type-specific genes. **(G)** GSVA showing differences in pathway activity among the major cell populations. **(H)** UMAP feature plots of representative cell-type marker genes. **(I)** Overall relative cell-type composition in the normal and tumor groups; this panel is descriptive and was not used as a cell-level test of statistical significance. **(J)** Relative cell-type composition across the 17 individual biological samples, illustrating inter-sample variation.

### 3.6 Identification of neuronal subpopulations

Neuronal cells were extracted and divided into 15 subclusters. MiloR analysis revealed clear differences in neuronal abundance between normal and tumor samples (**Figures 6A, B**). Based on neighborhood distribution, subclusters 1, 2, 3, 4, and 6 were classified as Normal_neurons, subcluster 8 as Tumour_neurons, and subcluster 0 as Special_neurons. The identified neuronal populations were observed across multiple biological samples rather than being driven by a single individual dataset. The remaining subclusters were grouped as Unrelated_neurons (**Figure 6C**). GSVA showed distinct pathway activities among these neuronal populations. Differences were observed in synaptic vesicle exocytosis, AMPA glutamate receptor activity, glutamate-gated calcium-channel activity, and morphogenesis-related pathways (**Figure 6D**).

**Figure 6:**
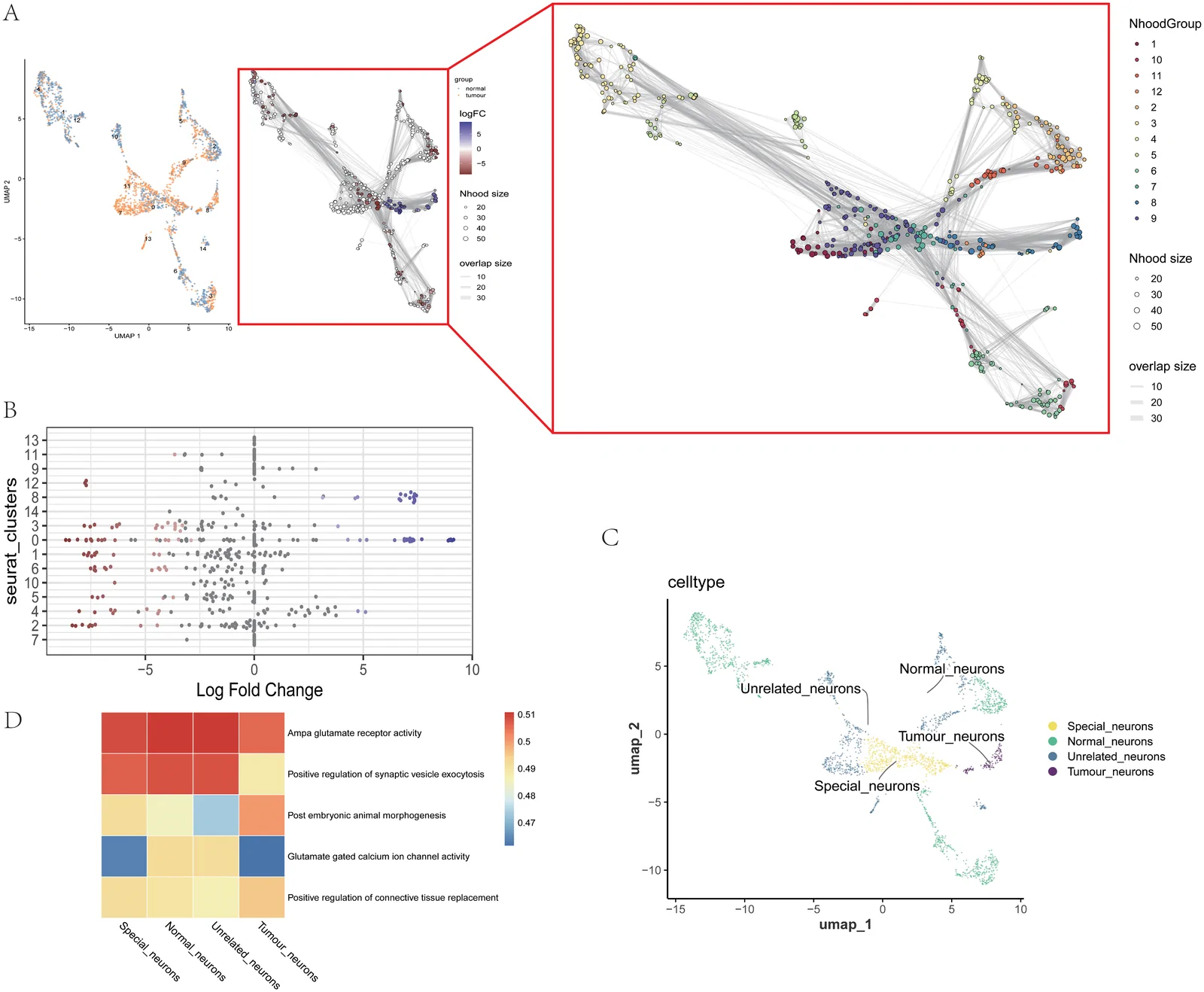
Differential abundance and functional characterization of neuronal subpopulations. **(A)** UMAP representation of neuronal clusters with MiloR neighborhoods overlaid on the embedding. **(B)** Differential abundance of neuronal neighborhoods between normal and tumor samples across the Seurat-defined clusters. **(C)** UMAP showing neuronal cells classified as Normal_neurons, Tumour_neurons, Special_neurons, or Unrelated_neurons according to their abundance patterns. **(D)** GSVA comparing pathway activity among the four neuronal populations.

### 3.7 Pseudotime organization of neuronal states

Monocle2 organized neuronal cells into five transcriptional states along an inferred pseudotime manifold (**Figures 7A–C**). The four neuronal populations showed different but partly overlapping distributions across this manifold. Normal_neurons and Tumour_neurons showed preferential distributions in different regions of the inferred transcriptional state space. The prognostic genes also showed heterogeneous expression patterns across the manifold (**Figure 7D**). EPHB6, RAB11FIP4, DMTN, and RPH3A were preferentially expressed in regions enriched for Normal_neurons, whereas PDIA4 showed higher expression in regions associated with the tumor-related neuronal state. BEAM analysis at the selected bifurcation identified gene modules associated with synaptic organization, membrane-potential regulation, exocytosis, glial differentiation, and trans-synaptic signaling (**Figure 7E**). These patterns were interpreted as differences among transcriptional states rather than evidence of lineage conversion.

**Figure 7:**
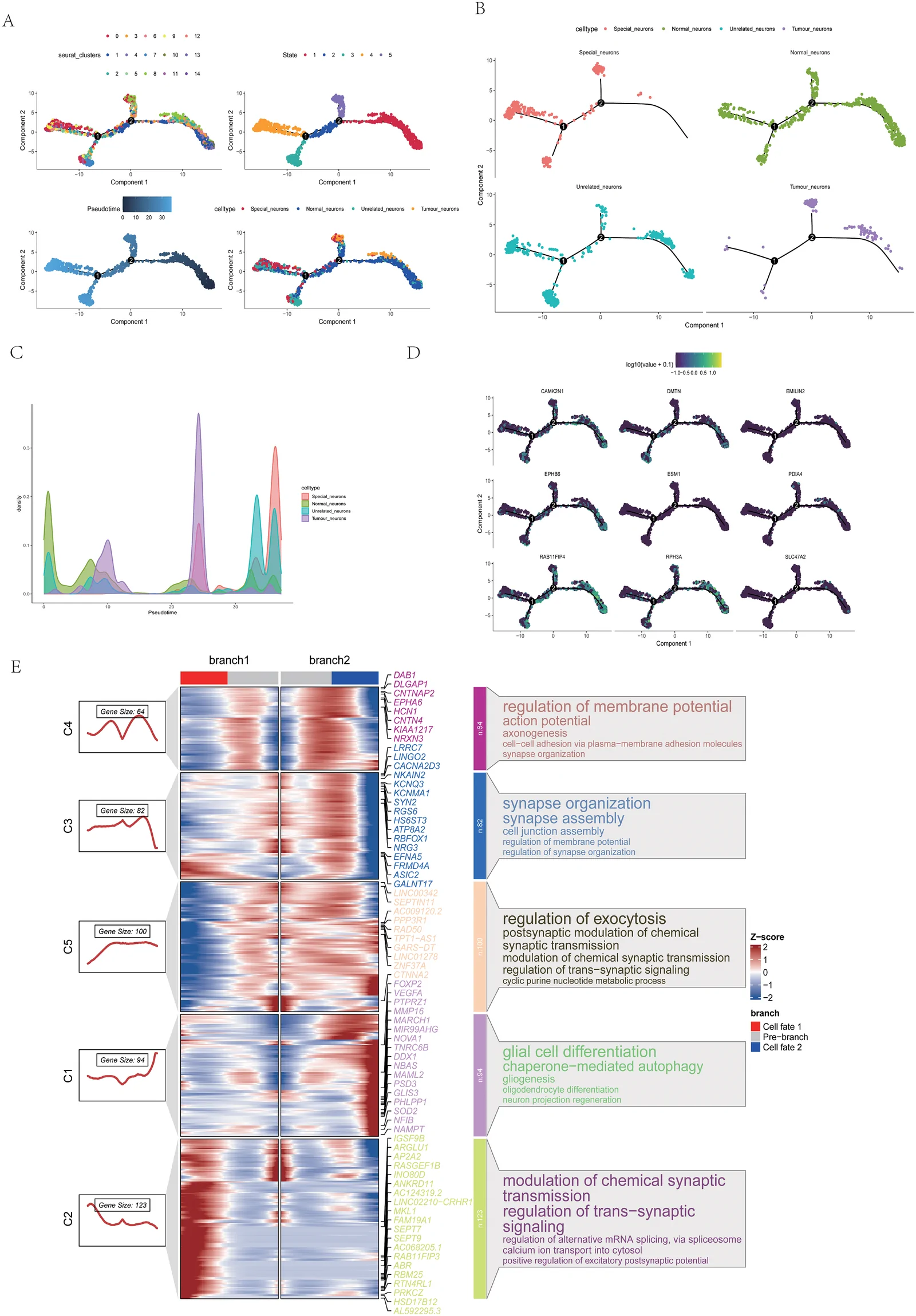
Pseudotime organization of neuronal transcriptional states. **(A)** Monocle2-derived pseudotime manifold showing the distribution of inferred neuronal transcriptional states. **(B)** Distribution of Normal_neurons, Tumour_neurons, Special_neurons, and Unrelated_neurons across the inferred manifold. **(C)** Density distribution of the four neuronal populations along pseudotime. **(D)** Expression patterns of the nine prognostic genes across the inferred pseudotime manifold. **(E)** BEAM analysis at the selected bifurcation point showing differential gene-expression patterns across the inferred branches and functional enrichment of the corresponding gene modules. The inferred pseudotime structure represents transcriptional relationships among neuronal states and does not establish developmental progression or lineage conversion.

### 3.8 Altered cell–cell communication in GBM

CellChat analysis revealed differences in incoming and outgoing signaling among neuronal populations, microglia, and GBM cells between tumor and normal samples (**Figures 8A–C**). Comparison of pathway-level information flow identified several altered signaling programs, including NRXN signaling (**Figure 8D**). NRXN communication networks differed between tumor and normal tissues (**Figures 8E, F**). Source–target analysis indicated predicted communication from neuronal populations toward GBM cells and microglia (**Figure 8G**). Ligand–receptor analysis further identified NRXN–NLGN family interactions contributing to the predicted communication network (**Figure 8H**).

**Figure 8:**
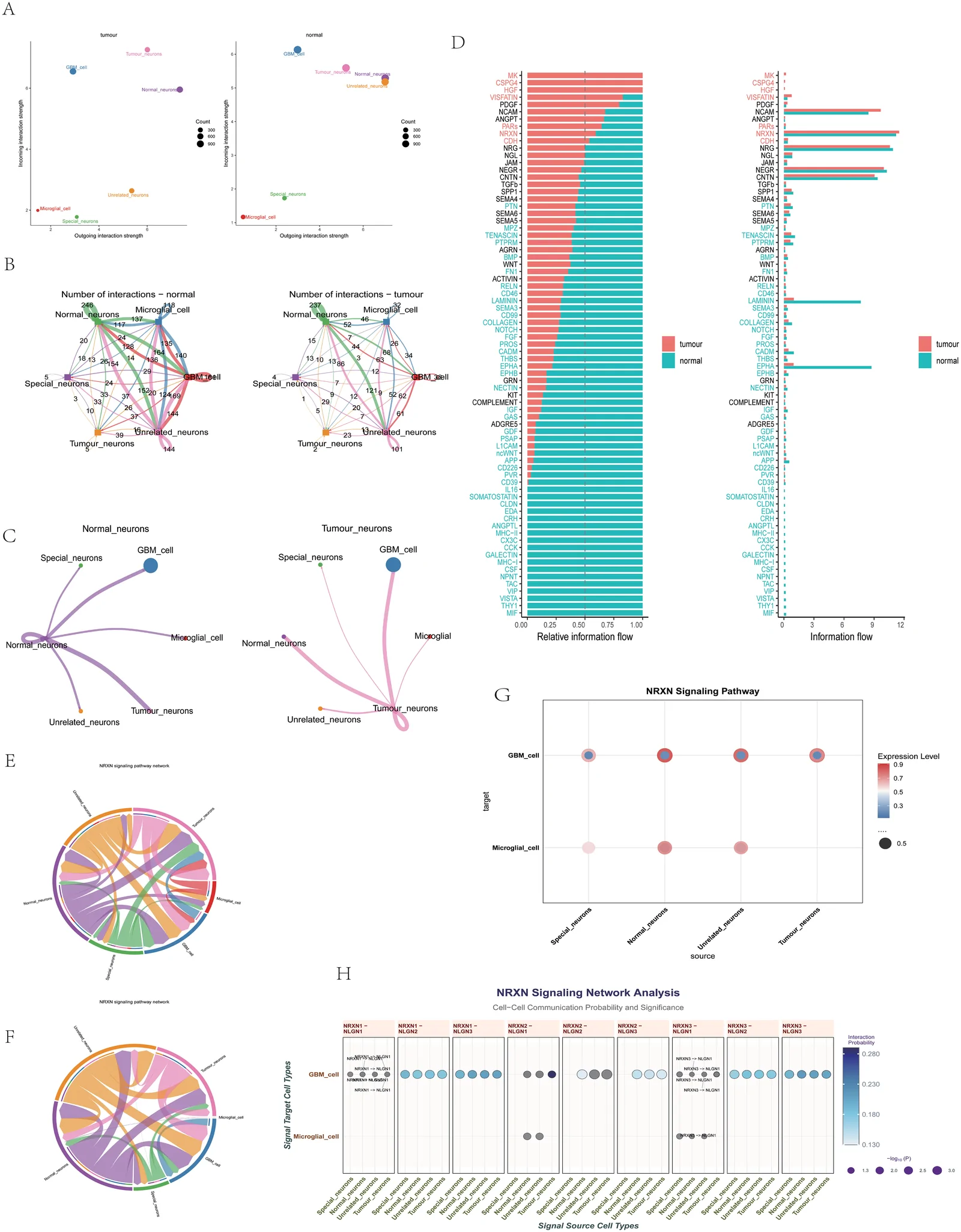
Cell–cell communication analysis of neuronal populations, microglia, and GBM cells. **(A)** Comparison of outgoing and incoming interaction strengths among the indicated cell populations in tumor and normal samples. **(B)** CellChat networks showing the number of predicted intercellular interactions in the normal and tumor groups. **(C)** Outgoing communication networks centered on Normal_neurons and Tumour_neurons in the tumor group. **(D)** Comparison of relative and absolute information flow across signaling pathways between tumor and normal samples. **(E, F)** Chord diagrams showing the NRXN signaling network in the tumor **(E)** and normal **(F)** groups. **(G)** Predicted source–target relationships within the NRXN signaling pathway, with neuronal populations as signaling sources and GBM cells or microglia as target populations. **(H)** Bubble plot showing the predicted NRXN–NLGN ligand–receptor pairs contributing to communication among neuronal populations, GBM cells, and microglia. Bubble color represents interaction probability, and bubble size reflects statistical significance.

### 3.9 Transcriptional regulatory programs in neuronal populations

SCENIC analysis showed distinct transcription-factor regulon activities among neuronal subpopulations (**Figures 9A, B**). HMGA2 and ONECUT2 were among the most active regulons in Tumour_neurons from tumor samples (**Figure 9C**). Regulatory networks were subsequently reconstructed for HMGA2, ONECUT2, NFIB, and KLF7, revealing distinct sets of predicted downstream target genes (**Figures 9D–G**).

**Figure 9:**
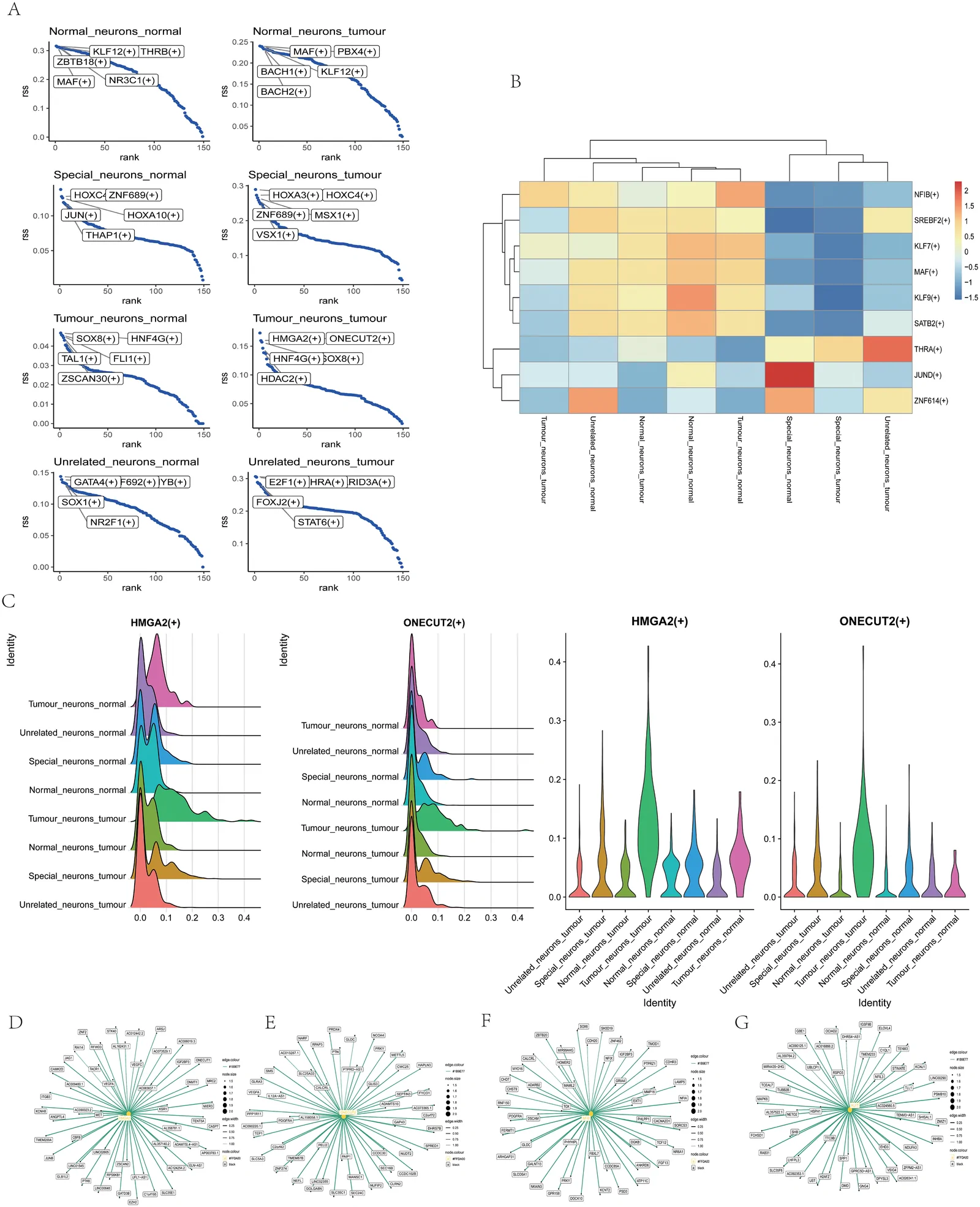
Transcription-factor regulon analysis of neuronal subpopulations. **(A)** Ranking of transcription-factor regulons according to regulon specificity scores across neuronal subpopulations from tumor and normal samples. **(B)** Heatmap showing the activity of selected transcription-factor regulons across the neuronal populations. **(C)** Distribution of HMGA2 and ONECUT2 regulon activity and expression among neuronal subpopulations. **(D–G)** Predicted regulatory networks of HMGA2 **(D)**, ONECUT2 **(E)**, NFIB **(F)**, and KLF7 **(G)**, showing their associated downstream target genes.

### 3.10 Complementary functional assessment of PDIA4 in GBM cells

PDIA4 protein expression was higher in U251 and U87 cells than in SVG p12 cells (**Figure 10A**). Transfection with PDIA4-targeting siRNA reduced PDIA4 protein and mRNA levels in both GBM cell lines (**Figures 10B, C**). PDIA4 depletion was accompanied by fewer invading cells and colonies, reduced wound closure reflecting altered motility-associated phenotype, and lower CCK-8 signals compared with the negative-control group (**Figures 10D–G**). These findings indicate that reduced PDIA4 expression is associated with impaired growth- and motility-related phenotypes in U251 and U87 cells.

**Figure 10:**
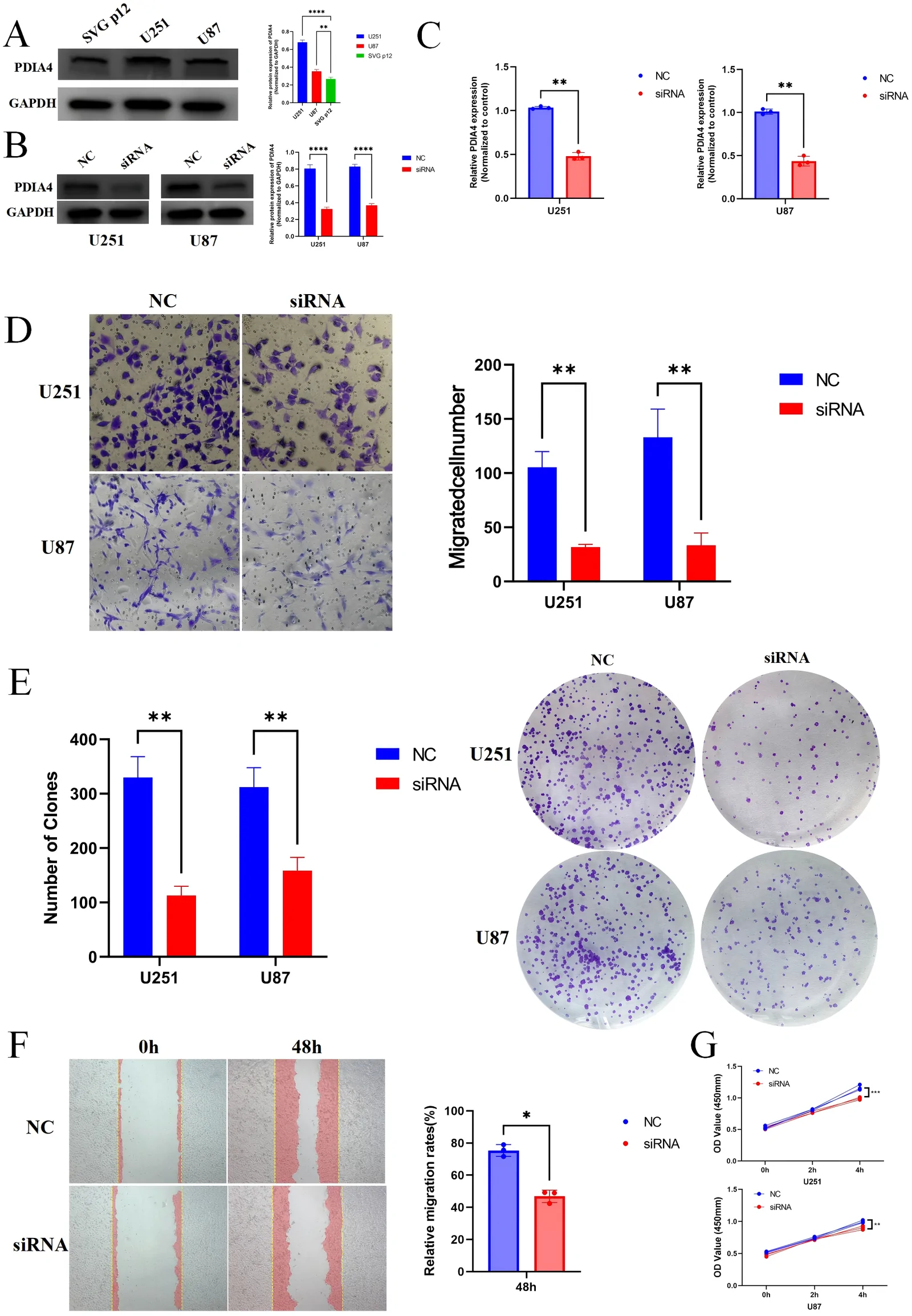
*In vitro* assessment of PDIA4 in GBM cells. **(A)** Western blot analysis of basal PDIA4 protein expression in SVG p12, U251, and U87 cells, with densitometric quantification normalized to GAPDH. **(B)** Western blot analysis and densitometric quantification of PDIA4 in U251 and U87 cells transfected with negative-control siRNA (NC) or PDIA4-targeting siRNA. **(C)** Relative PDIA4 mRNA expression measured by qRT-PCR after siRNA transfection. **(D)** Representative Transwell images and quantification of invaded cells. **(E)** Representative colony-formation images and quantification of colony numbers. **(F)** Representative wound-healing images obtained at 0 and 48 h and quantification of wound closure. Because wound closure may reflect both cell motility and growth, this assay was interpreted as a composite phenotype rather than a migration-specific measurement. **(G)** CCK-8 measurements in NC and PDIA4-silenced U251 and U87 cells. Quantitative data are presented as mean ± SD from three independent biological experiments. *P < 0.05, **P < 0.01, ***P < 0.001, and ****P < 0.0001.

## 4 Discussion

Glioblastoma is sustained by extensive cellular heterogeneity and continuous interactions between malignant cells and the surrounding brain microenvironment. Single-cell and spatial profiling studies have increasingly shown that the biological behavior of GBM cannot be explained solely by tumor-cell-intrinsic programs, as immune, vascular, glial, and neuronal components all contribute to tumor evolution and therapeutic resistance (1, 2, 10, 11). In this study, we integrated bulk transcriptomics, WGCNA, machine-learning-based survival modeling, immune deconvolution, and single-cell RNA sequencing to characterize prognostic features across different cellular compartments. Nine genes were retained in the final prognostic model, several of which showed clear enrichment in neuronal populations. Further analyses identified a tumor-associated neuronal state with altered abundance, synaptic activity, intercellular communication, and transcription-factor regulation. In parallel, functional experiments showed that PDIA4 contributes to malignant phenotypes in GBM cells.

The nine-gene signature consisted of PDIA4, CAMK2N1, RPH3A, RAB11FIP4, DMTN, EPHB6, SLC47A2, ESM1, and EMILIN2. These genes were not restricted to a single cellular compartment. Their distribution across endothelial, neuronal, microglial, and other cell populations suggests that part of the prognostic information captured in bulk tissue reflects differences in the tumor ecosystem rather than only differences among malignant cells. This interpretation is consistent with recent work showing that GBM transcriptional architecture is organized across multiple malignant and non-malignant layers (1). Longitudinal single-cell studies have also demonstrated that treatment and progression can substantially reshape these states without requiring uniform acquisition of new genomic alterations (2, 10). The enrichment of the nine genes in immune, proliferative, and neuronal pathways therefore fits well with the concept that prognosis in GBM is influenced by both intrinsic tumor programs and microenvironmental composition.

The immune landscape showed clear differences between tumor and normal samples. M2 macrophages, NK-cell subsets, mast cells, neutrophils, M1 macrophages, follicular helper T cells, and CD8 T cells were among the populations that differed between groups. In addition, PDIA4, SLC47A2, EMILIN2, and ESM1 were associated with several immune-cell fractions. This pattern is consistent with the well-established predominance of heterogeneous myeloid populations and dysfunctional lymphocyte states in GBM (3, 12–14). Yeo et al. reported progressive remodeling of the immune compartment toward increasingly suppressive myeloid states during GBM evolution (3), while Ravi et al. showed that glioma-associated myeloid cells can promote T-cell dysfunction through IL-10-mediated signaling (13). Spatial studies further indicate that these immune states are unevenly distributed within individual tumors (12). Our results add a prognostic-gene dimension to this picture, although the correlations should not be interpreted as direct regulatory relationships. In particular, the negative association between PDIA4 and activated NK cells, together with its positive association with resting NK cells, is suggestive but does not establish that PDIA4 directly alters NK-cell abundance or function.

The single-cell analysis placed several prognostic genes within neuronal populations. EPHB6, DMTN, RAB11FIP4, and RPH3A were preferentially expressed in neurons, whereas CAMK2N1 was mainly detected in microglia. MiloR analysis further separated the neuronal compartment into normal-associated, tumor-associated, special, and unrelated populations, with tumor-associated neurons enriched in tumor samples. This observation is notable in the context of recent work in cancer neuroscience. GBM cells are now known to interact functionally with neuronal circuits rather than simply growing within neural tissue (5, 6, 15–19). In patients, stronger functional connectivity between glioblastoma and surrounding cortical networks has been associated with shorter survival (5). Experimental studies have shown that neuronal activity can stimulate glioma growth and invasion, while glioma cells can in turn remodel local neural and tumor-cell networks (6, 15, 16). A neural epigenetic signature has also been linked to outcome in high-grade glioma (17). The enrichment of tumor-associated neuronal populations in our dataset therefore adds an independent transcriptomic layer to a biological phenomenon already supported by functional and clinical studies.

The pseudotime analysis suggested that neuronal states were accompanied by marked transcriptional differences. Normal-associated and tumor-associated neuronal populations occupied partially distinct regions of the inferred transcriptional manifold, suggesting differences in neuronal state programs rather than a developmental sequence. Genes involved in membrane potential, synaptic organization, exocytosis, and trans-synaptic signaling changed along this trajectory. These patterns are compatible with neuronal remodeling in the tumor microenvironment, but they should not be taken as evidence that mature normal neurons physically differentiate into tumor-associated neurons. Pseudotime analysis captures transcriptional relationships between cell states rather than direct lineage conversion. Even so, the observed shift is biologically plausible. Spatial profiling of infiltrating GBM has shown increased expression of neurodevelopmental, synaptic, and Notch-related programs at the tumor–brain interface (18), and direct mapping of neuron–tumor networks has demonstrated widespread integration of GBM cells into neuronal circuitry (19). Our data therefore point to altered neuronal states associated with tumor tissue rather than a proven lineage transition.

CellChat analysis identified NRXN signaling as one of the communication programs that differed between tumor and normal samples. Predicted interactions involving NRXN and NLGN family members linked neuronal populations with GBM cells. Given the established role of neurexins and neuroligins in synaptic adhesion, this finding is relevant to the growing evidence that glioma cells exploit components of normal neural communication. At the same time, the functional significance of this pathway in glioma remains uncertain. Neuron–GBM connectivity itself has been shown to promote invasion, growth, and treatment resistance (19). In contrast, Liao et al. reported that exogenous NRXN1 promoted neuron-like differentiation and reduced malignant features in glioma models (20). These apparently different observations suggest that the biological effect of NRXN signaling may depend on cellular context, isoform usage, interacting partners, and the mode of cell contact. For this reason, our CellChat result is best viewed as a candidate communication pathway rather than evidence of a confirmed tumor-promoting NRXN–NLGN mechanism.

The relationship between neuronal remodeling and immune regulation may also be relevant. Nejo et al. recently showed that GBM regions with stronger neuronal connectivity display regional immunosuppression and enrichment of anti-inflammatory tumor-associated macrophages (7). Our study independently identified both neuronal-state differences and changes in immune-cell composition. Although we did not directly test whether the neuronal populations identified here regulate immune cells, these findings raise the possibility that neuronal and immune changes may occur within the same tumor niches. Spatial transcriptomics, multiplex imaging, or spatial proteomics would be useful for determining whether tumor-associated neurons, PDIA4-high cells, and specific immune populations co-localize in human GBM tissue.

SCENIC analysis revealed additional heterogeneity at the transcriptional-regulatory level. HMGA2 and ONECUT2 were among the prominent regulons in tumor-associated neurons, whereas NFIB and KLF7 were associated with distinct predicted regulatory networks. Several components identified in these analyses have previously been implicated in glioma biology. HMGA2 has been reported to promote glioma malignancy through transcriptional repression of IGFBP3 (21). Among the predicted downstream components, TUBB2B has been shown to promote glioma migration and invasion through interaction with vimentin (22), whereas ZBTB20 has been reported to suppress GBM growth through the TET1/FAS/caspase-3 pathway (23). These published observations support the biological relevance of individual components identified in our analysis, but they do not validate the specific transcription-factor–target relationships inferred here. The SCENIC-derived regulatory networks should therefore be regarded as computational predictions pending experimental perturbation or binding studies.

Among the prognostic genes, PDIA4 was selected for experimental validation because of its elevated expression in GBM and its association with adverse tumor-related features in the computational analyses. Basal PDIA4 protein expression was higher in U251 and U87 cells than in the glial control cell line. siRNA-mediated depletion reduced both PDIA4 mRNA and protein levels and was accompanied by weaker colony formation, invasion, and wound closure. CCK-8 measurements were also lower after PDIA4 silencing, indicating reduced metabolic activity/viability under the assay conditions. These results are in line with previous experimental studies. Wang et al. showed that PDIA4 promotes GBM progression through the PI3K/AKT/mTOR pathway (8). Tu et al. further reported that PDIA4 participates in endoplasmic-reticulum stress adaptation and angiogenesis, with XBP1-dependent PDIA4 induction promoting VEGFA secretion and resistance to anti-angiogenic therapy (9). The present findings therefore support PDIA4 as a functionally relevant molecule in GBM rather than only a prognostic transcript. These findings should be considered in the context of the existing PDIA4 literature. Our experiments were not designed to re-establish PDIA4 as a novel GBM driver, since its association with malignant growth, PI3K/AKT/mTOR signaling, ER stress, and angiogenesis has already been reported. Rather, PDIA4 was selected as one component of the nine-gene prognostic signature for complementary functional assessment. The distinction of the present study lies primarily in linking this signature to specific cellular compartments of the GBM ecosystem, particularly neuronal populations, and in integrating neuronal differential abundance, transcriptional state, predicted intercellular communication, and regulatory-network analyses.

The relationship between PDIA4 and the neuronal findings deserves a more cautious interpretation. PDIA4 increased in regions of the neuronal pseudotime trajectory associated with the tumor-related state, whereas its knockdown produced clear phenotypic effects in established GBM cell lines. These two observations do not demonstrate that neuronal signaling drives PDIA4 expression, nor do they show that PDIA4 functions downstream of NRXN–NLGN signaling. At present, it is more appropriate to consider PDIA4 as a marker of a tumor-associated transcriptional program that also has a tumor-cell-intrinsic role. This interpretation is compatible with the known function of PDIA4 in endoplasmic-reticulum stress responses, particularly in the context of the hypoxic, metabolic, and proteotoxic stress characteristic of GBM. Whether neuronal stimulation or direct neuron–GBM contact alters PDIA4 expression remains an open question.

Several limitations should be noted. First, complete annotation of established GBM prognostic factors, including IDH mutation status, MGMT promoter methylation, and molecular subtype, was not available for all TCGA samples. Therefore, the present analysis cannot determine whether the nine-gene signature provides prognostic information independent of these established molecular factors, and validation in well-annotated independent clinical cohorts will be required. In addition, although ComBat correction was applied to reduce technical variation between TCGA and GTEx datasets, residual differences related to tissue composition, anatomical origin, and donor characteristics cannot be completely excluded. Therefore, some bulk transcriptomic differences may reflect differences in cellular composition rather than tumor-specific transcriptional regulation. The neuronal states identified by MiloR and pseudotime analysis represent transcriptomically defined populations, and their reproducibility will require confirmation in additional patient-level single-cell datasets. Similarly, CellChat and SCENIC provide computational predictions based on transcriptomic information and should not be considered direct evidence of ligand–receptor signaling or transcription-factor binding. In particular, the predicted NRXN–NLGN interactions and neuronal regulatory programs were not experimentally validated at the protein or functional level in the present study.

The functional experiments also have several limitations. PDIA4 was examined in established U251 and U87 cell lines using a single targeting siRNA. Although knockdown was confirmed at both the mRNA and protein levels and consistent effects were observed across multiple phenotypic assays, additional validation using independent targeting sequences, rescue experiments, patient-derived GBM models, and orthotopic *in vivo* systems would provide stronger evidence for target specificity and translational relevance. Furthermore, the provenance of commonly used U87 stocks has been questioned, and although similar PDIA4-associated phenotypes were observed in both U251 and U87 cells, confirmation in authenticated patient-derived GBM models will be important. The SVG p12 comparison should also be interpreted as a non-neoplastic glial reference rather than a fully matched normal brain control. Finally, the CCK-8 assay reflects cellular metabolic activity rather than direct cell division, and the predicted NRXN–NLGN pathway, neuronal transcription-factor programs, PDIA4 expression changes, and immune alterations remain biologically associated observations rather than a fully established causal mechanism.

Taken together, our results support a model in which the prognostic landscape of GBM reflects contributions from multiple cellular compartments, including malignant, immune, and neuronal populations. Tumor-associated neuronal states showed altered abundance, synaptic programs, and regulatory activity, while NRXN–NLGN signaling emerged as a candidate route of neuron–GBM communication. PDIA4, identified through the prognostic analyses, was further supported by functional experiments showing that its depletion reduced clonogenicity, invasion, wound closure, and CCK-8 metabolic activity readouts in GBM cells. These findings provide a basis for further studies addressing how neuronal remodeling, tumor-cell stress responses, and immune organization interact within the GBM microenvironment.

## 5 Conclusion

In summary, this study integrates bulk transcriptomics, prognostic modeling, and single-cell analysis to connect a nine-gene prognostic signature with the cellular organization of GBM. The results highlight neuronal heterogeneity and a tumor-associated neuronal state as potentially relevant components of the GBM prognostic ecosystem and identify candidate neuron–GBM communication and regulatory programs for further investigation. PDIA4 knockdown provides complementary functional support for one component of the prognostic signature, but its established role in GBM and the limitations of the present cell-line experiments should be considered when interpreting these findings. Further validation in independent patient cohorts, spatially resolved tissue, and patient-derived experimental models will be required.

## Data Availability

The original contributions presented in the study are included in the article/supplementary material. Further inquiries can be directed to the corresponding author/s.
